# [Retracted] Correlation of SOX9 and NM23 genes with the incidence and prognosis of prostate cancer

**DOI:** 10.3892/ol.2024.14324

**Published:** 2024-03-04

**Authors:** Ming Xi, Song Wan, Wei Hua, Yulin Zhou, Wencong Jiang, Jianxin Hu

Oncol Lett 17: 2296–2302, 2019; DOI: 10.3892/ol.2018.9828

Following the publication of this paper, it was drawn to the Editor's attention by a concerned reader that the si-SOX9 cell migration assay data shown in Fig. 3A on p. 2300 were strikingly similar to data in a different article by different authors at different research institutes that were already under consideration for publication [Lian Y, Xiong F, Yang L, Bo H, Gong Z, Wang Y, Wei F, Tang Y, Li X, Liao Q *et al*: Long noncoding RNA AFAP1-AS1 acts as a competing endogenous RNA of miR-423-5p to facilitate nasopharyngeal carcinoma metastasis through regulating the Rho/Rac pathway. J Exp Clin Cancer Res 37: 253, 2018].

Owing to the fact that some of the data in the above article were already under consideration for publication prior to its submission to *Oncology Letters*, the Editor has decided that this paper should be retracted from the Journal. The authors were asked for an explanation to account for these concerns, but the Editorial Office did not receive a reply. The Editor apologizes to the readership for any inconvenience caused.

